# Cortical Bone Mechanical Properties Are Altered in an Animal Model of Progressive Chronic Kidney Disease

**DOI:** 10.1371/journal.pone.0099262

**Published:** 2014-06-09

**Authors:** Christopher L. Newman, Sharon M. Moe, Neal X. Chen, Max A. Hammond, Joseph M. Wallace, Jeffry S. Nyman, Matthew R. Allen

**Affiliations:** 1 Department of Anatomy and Cell Biology, Indiana University School of Medicine, Indianapolis, Indiana, United States of America; 2 Division of Nephrology, Department of Medicine, Indiana University School of Medicine, Indianapolis, Indiana, United States of America; 3 Roudebush VA Medical Center, Indianapolis, Indiana, United States of America; 4 Weldon School of Biomedical Engineering, Purdue University, West Lafayette, Indiana, United States of America; 5 Department of Biomedical Engineering, Indiana University—Purdue University, Indianapolis, Indiana, United States of America; 6 Department of Orthopaedic Surgery and Rehabilitation and Vanderbilt Center for Bone Biology, Vanderbilt University Medical Center, Nashville, Tennessee, United States of America; 7 Department of Veterans Affairs, Tennessee Valley Healthcare System, Nashville, Tennessee, United States of America; University of California Davis, United States of America

## Abstract

Chronic kidney disease (CKD), which leads tocortical bone loss and increasedporosity,increases therisk of fracture. Animal models have confirmed that these changes compromise whole bone mechanical properties. Estimates from whole bone testing suggest that material properties are negatively affected, though tissue-level assessmentshavenot been conducted. Therefore, the goal of the present study was to examine changes in cortical bone at different length scales using a rat model with theprogressive development of CKD. At 30 weeks of age (∼75% reduction in kidney function), skeletally mature male Cy/+ rats were compared to their normal littermates. Cortical bone material propertieswere assessed with reference point indentation (RPI), atomic force microscopy (AFM), Raman spectroscopy,and high performance liquid chromatography (HPLC). Bones from animals with CKD had higher (+18%) indentation distance increase and first cycle energy dissipation (+8%) as measured by RPI.AFM indentation revealed a broader distribution of elastic modulus values in CKD animals witha greater proportion of both higher and lower modulus values compared to normal controls. Yet, tissue composition, collagen morphology, and collagen cross-linking fail to account for these differences. Though the specific skeletal tissue alterations responsible for these mechanical differences remain unclear, these results indicate that cortical bone material properties are altered in these animals and may contribute to the increased fracture risk associated with CKD.

## Introduction

Chronic kidney disease—mineral and bone disorder (CKD-MBD) is characterized by hyperphosphatemia, secondary hyperparathyroidism, and an increased risk offractures[1]–[3].Unlike osteoporosis, CKD-MBD appears to have a preferential impact on cortical bone, leading to reduced bone mass and increased porosity [4]–[6].These effects likely underlie the increased fracture risk observed in patients with CKD[7]–[9].

Whole bone (structural)mechanical properties are dependent upon a number of variables [10]–[11]. While bone mass is a major determinant, both the distribution of bone and its material properties (inherent physical and chemical properties) also play crucial roles. Most biomechanical studies in rodent models of CKD have focused on structural mechanical properties, employing three-point bending or dynamic mechanical analysis (DMA) [12]–[18]. These studies indicate that the bending and viscoelastic properties of bone are compromised in animals with CKD. Specifically, DMA indicates that diseased animals have lower storage modulus (a measure of stiffness) and tan delta (a measure of energy dissipation) [14]–[15], while three point bending studies indicate that ultimate load, stiffness, and energy to failure are lower in CKD animals [12].

While there is an increasing awareness of the importance of bone quality in CKD [19], few studies have explicitly examined material properties in animal models[14]–[15], [20]. Material properties can be estimated from whole bone mechanical tests using standardengineering equations that account for whole bone structure and geometry.Because these estimates assume that skeletal tissue is homogeneous, isotropic, and linearly elastic, direct measures ofmaterial properties in bone would provide additionalinsight into how the disease is affecting fracture resistance. Therefore, the goal of the present study was to examine material-level changes in cortical bone at several length scales using a rat model with the progressive development of CKD. Specifically, we hypothesized that CKD adversely impacts cortical bone material properties as determined by material-level mechanical testing and assessments of bone composition and collagen morphology.

## Materials And Methods

### Animal Model

The current study utilized a slowly progressive animal model of CKD, the Cy/+ rat. Cy/+ ratsare characterized by autosomal dominant polycystic kidney disease [21]. These animals have a mutation (R823W) in *Anks6*, a gene that codes for the protein SamCystin. Currently, the function of this protein is unknown, and the specific role of this mutation in the development of polycystic kidney disease is unclear. Aside from its expression in the kidney, however, little is known about its role in the cell [22].Unlike most other PKD-related proteins, though, SamCystin does not localize to the primary cilia of kidney cells [23]. While there is no known human disease associated with this gene, thespontaneous onset of disease provides a helpful phenotypic model of human CKD [21].Unlike the more common surgical models[24], Cy/+ rats display a gradual onset of disease.And unlike most genetic models[25]–[26], they exhibit a slow enough progression that bone disease does not begin to occur until after skeletal maturity.

Skeletal tissuefrom animals in a previous study wasutilized [12]. All animals were fed a casein diet (Purina AIN-76A; 0.7% Pi) to increase phosphorus availability in order to produce a more consistent kidney disease phenotype.Fresh frozen tibiaefrom 30-week-old male Cy/+ ratsand their age-matched non-affected littermates were assessed mechanically, compositionally, and morphologically at several length scales.Fresh frozen femora were used for collagen cross-linking analyses. Blood was collected at the end of the experiment for biochemical analyses (previously reported in [12]).All procedures were conducted under the approval of Indiana University School of Medicine Institutional Animal Care and Use Committeeprotocol # 10479.

### Reference Point Indentation

Tibiae were thawed to room temperature and soaked overnight in phosphate-buffered saline. The anteromedial surface of the proximal diaphysis of the tibia was assessed using reference point indentation (RPI) (Biodent Hfc, Active Life Scientific, Santa Barbara, CA). The reference probe, which housed a BP2 test probe, waslowered vertically, normal to the surface, until itrested on the surface of the bone.In order to stabilize the unit, a reference force of ∼13 N was applied before each measurement was initiated.Each test included a series of 10 cycles at 2 Hz to a force of 10 N. Bones were maintained in a hydrated state throughout the test. Five locations per sample, each ∼2 mm apart, were indented. Raw data from the RPI analysis software (version 2.0) were imported into a customized MATLAB code (Mathworks) designed to provide cycle-by-cycle data for each test [27], from which first cycle unloading slope, indentation distance increase, first cycle energy dissipation, creep indentation distance, first cycle indentation distance, total indentation distance, and total energy dissipation were calculated for each test. All five tests from each animal were averaged to produce a single value for each variable.

### Tissue Composition

Raman spectroscopy was performed using a LabRAM HR 800 Raman Spectrometer (HORIBA JobinYvon, Edison, NJ) connected to a BX41 microscope (Olympus, Tokyo, Japan). A 660 nm laser was focused on the bone surface using a 50X objective to a spot size of ∼10 µm. Five locations were imaged ∼1 mm apart on the anteromedialmid-diaphysiswith five 20 second acquisitions at each location as previously published [28]. A five point linear baseline correction was applied in LabSpec 5 (HORIBA JobinYvon). Using OriginPro 8.6 (OriginLab, Northampton, MA), a single Gaussian peak was fit to the PO_4_^3-^ν1 peak, and the areas under the PO_4_^3-^ν1, CO_3_^2-^ν1, and Amide I peaks were calculated at each location. Type B carbonate substitution was found by the band area ratio of CO_3_^2-^ν1/PO_4_^3-^ν1. The degree of matrix mineralization was determined by the band area ratios of PO_4_^3-^ν1/Amide I. Mineral maturity (crystallinity) was determined by the inverse of the full width at half maximum (FWHM) of the PO_4_^3-^ν1 peak.

### AFM Indentation

The anteromedial portion of the mid-diaphysis used above was polished with a 3 µm polycrystalline water-based diamond suspension in order to create a flat region for testing. Nanoindentation was performed using a BioScope Catalyst atomic force microscope (Bruker, Santa Barbara, CA), operating in peak force tapping mode using previously published methods [29]. Indentations were performed using a polycrystalline diamond probe (NaDia ND-DYC series; Advanced Diamond Technologies, Inc.) with a measured spring constant of 29.25 N/m. Four locations per sample were indented and, at each location (20 µm×20 µm grid), 49 indentationswere performed. Samples were loaded to 200 nN with force-separation curves acquired from each indentation. Within each location, indentations were spaced about 2 µm apart in order to avoid interactions from neighboring indentations. In total, 196 indentations were performed for each sample. The indentation elastic modulus was calculated from 5% to 95% of the withdrawal curve using the classic Hertz model of contact between a rigid sphere and an elastic half space because the indentation depth is much smaller than the radius of curvature of the probe [29]. The indentation elastic modulus was determined from the following equation: E = 3F(1-v∧2)/(4r∧(1/2) δ∧(3/2)) where E is the indentation elastic modulus, F is the indentation force, v is the Poisson's ratio of the sample (assumed to be 0.35), r is the tip radius (nominal radius of 50 nm, with the same probe used for all samples), and δ is the indentation depth. All of the individual indentations were averaged to produce a single value for each animal, though individual tests were used for distribution comparisons among the groups.

### Collagen Morphology

Following AFMindentation, the polished surface was partially decalcified by soaking the bones in 0.5 M EDTA for 25 minutes followed by five minutes of sonication in a water bath. This process was repeated five times for each sample. For imaging, RTESPA probes were used (Bruker; radius nominally 8 nm, spring constant  = 40 N/m). The scan size was set at 3.5 µm with 512×512 pixels and a scan rate of 0.5 lines/s. For measurements of collagen morphology, four locations were imaged per sample, and 10 to 15 fibrils were measured at each location. 40 to 50 fibrils per sample were averaged to produce a single value for each animal, though the individual tests were used for distribution comparisons. Using SPIP 5.1.10 (Image Metrology, H∅rsholm, Denmark),D-periodic spacing was calculated using2D Fast Fourier Transformations (2D FFTs) as previously described [28].

### Collagen Cross-Linking

Segments of bone (∼3 mm in length) from the proximal femoral diaphysis were fully demineralized in 20% EDTA (0.68 M, pH 7.4). Approximately 10 mg of demineralized bonewerehydrolyzedin6 N HCl (∼10 µL per 1 mg) at 110°C for 20 to 24 hours. After evaporating the acidusing a SpeedVAC centrifuge with coldtrap, each hydrolysate was resuspended in ultrapure water, split into two equal portions, and dried. Half the residue was resuspended in ultrapure water with an internal standard (5×10^−6^ g/L pyridoxine). The solution was filtered and diluted with 0.05% heptafluorobutyric acid in 10% acetonitrile, and 50 µL of each hydrolysate were assayed by a high performance liquid chromatography (HPLC) system (Beckman-Coulter System Gold 168) with a silica-based column (Waters Spherisorb). Standards with varying concentrations of pyridinoline (Pyd) (Quidel),deoxypyridinoline (Dpd) (Quidel),pentosidine(PE) (International Maillard Reaction Society), and a constant amount of pyridoxinewere also assayed. Using a Waters 2475 fluorescence detector (excitation/emission of295/400 nm for Pyd and Dpd and 328/378 nm for PE), chromatograms were recorded to determine the amount of each crosslink. These amounts were then normalized by collagen content, which was determined from the other half of each hydrolysate by another HPLC assay [30]. Briefly, withα-amino-butyric acid (α-ABA) included as an internal standard, the amino acids were subjected to derivatization withphenyl isothiocyanate (PITC). Along with standards of varying concentrations of hydroxyproline (Sigma) and proline (Sigma)and a constant amount of α-ABA, the derivatized samples were resuspendedin a buffer solution of 5% acetonitrile in 5 mM disodium phosphate. Upon injecting 50 µLof this sample, chromatograms were generated with a UV detector (Beckman-Coulter System Gold 168). The calculated mass of hydroxyproline was then multiplied by 7.5 (assuming 13–14% of type I collagen by mass) and divided by the molecular weight of collagen (30,000 Da) [31], thereby giving crosslink concentration as mol/mol of collagen.

### Statistical Analysis

All analyses were performed using SPSS software. Comparisons between groups were made with Student's t-tests (assumptions validated by Shapiro-Wilk and Levene tests). When non-normal distributions or unequal variances were present, comparisons were made using Wilcoxon ranked-sum tests and unequal variance t-tests, respectively. Distributions included all measures from each individual, and comparisons were made using Kolmogorov-Smirnov tests. *A priori* α-levels were set at 0.05 to determine significance.

## Results

### Animal Model

**Figure 1:**
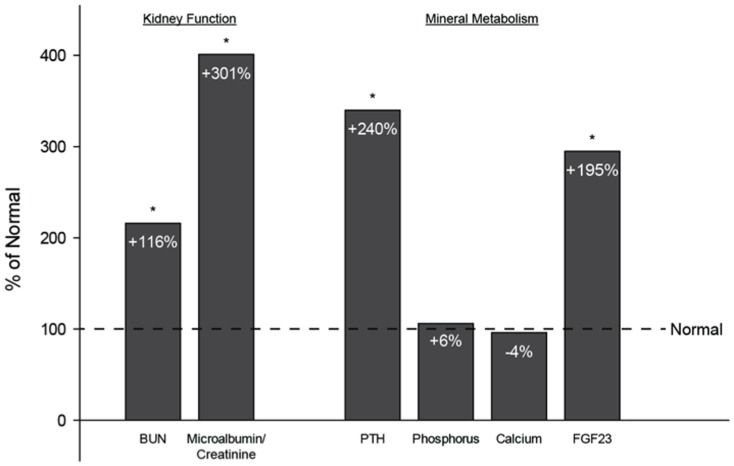
Biochemical assessment of kidney function and mineral metabolism. These previously published data (Allen *et al*., 2013) show abnormalities in kidney function and mineral metabolism resulting from hyperparathyroidism in the animals utilized in the current work. Data are presented as a percentage of non-affected normal animals with (*) representing statistical significance.

**Figure 2:**
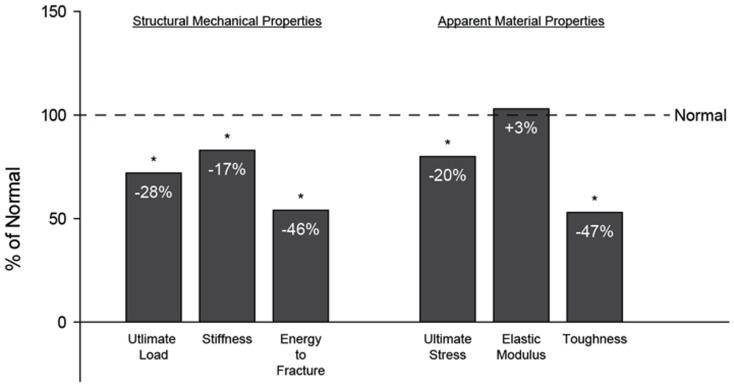
Structural mechanical properties and estimated material properties. These previously published data (Allen *et al*., 2013) show compromised whole bone mechanical properties from femoral 3-point bending and apparent material-level mechanical properties derived from standard beam bending equations in the animals utilized in the current work. Data are presented as a percentage of non-affected normal animals with (*) representing statistical significance.

Details about the phenotype of these animals have been previous published [12]. Briefly, measures of kidney function, including BUN (+116%) and the albumin-to-creatinine ratio (+301%), weresignificantly higher in Cy/+ animals compared to the normal controls. Similar to what is observed in humans with CKD, there were no differences between groups for phosphorus or calcium levels, but both serum PTH (+240%) and FGF23 (+195%) weredrastically higher(**FIGURE 1**).Cy/+ animals had higher numbers of osteoclasts and higher levels of bone remodeling. Using three-point bending, they exhibited lower ultimate load (−28%), stiffness(−17%), and energy to fracture (−46%). Estimates of material properties indicate that they had lower ultimate stress (−20%) and toughness (−47%) (**FIGURE 2**)[12].

### Reference Point Indentation

**Figure 3:**
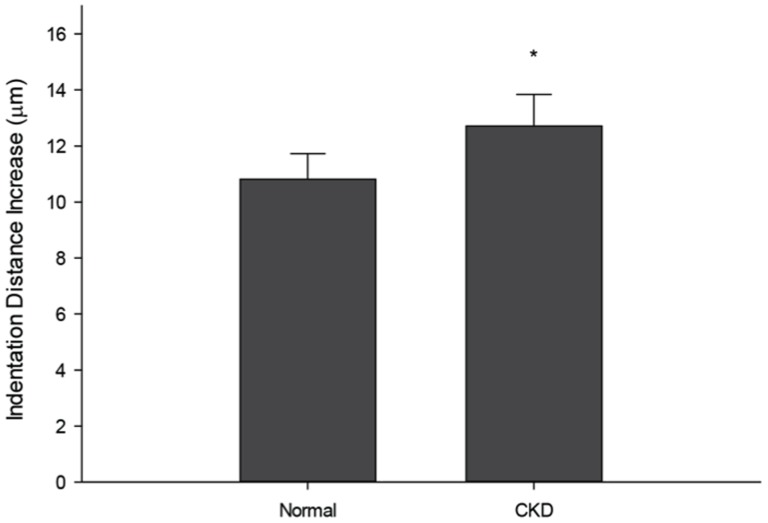
Microindentation reveals that CKD skeletal tissue is less able to resist damage. Using reference point indentation (RPI), the indentation distance increase (IDI) was found to be significantly higher in CKD animals compared to normal. These data indicate that the mechanical integrity of the bone is less able to resist microscale damage formation and propagation. Data are presented as mean and standard deviation. *p<0.05 versus normal controls.

Indentation distance increase (IDI)provides an assessment of the change in depth between the first cycle and the final cycle. Cy/+ animals had significantly higher IDI (+18%), indicating that the tissue ismore prone to damage under the same applied load (**FIGURE 3**). The amount of energy dissipated during the first cycle was also significantlyhigher in animals with CKD (+8%). While the first cycle creep indentation distance (+18%) was higher in Cy/+ animals, there was no difference in microstructural stiffness (first cycle unloading slope)between the two groups.No differences were noted in first cycle indentation distance, total indentation distance, or total energy dissipation (**Table 1**).

**Table 1:** Mechanical properties from microindentation and nanoindentation.

**RPI**	**Normal (n = 6)**	**Cy/+ (n = 6)**	**p-values**
First Cycle Indentation Distance (µm)	83.38±3.15	88.24±5.45	*0.088
First cycle Energy Dissipation (µJ)	275.53±14.00	297.94±19.35	* **0.044**
First Cycle Unloading Slope (N/µm)	0.47±0.02	0.45±0.03	*0.113
First Cycle Creep Indentation Distance (µm)	6.08±0.43	7.17±0.95	* **0.028**
Indentation Distance Increase (µm)	10.81±0.92	12.71±1.13	* **0.009**
Total Indentation Distance (µm)	89.39±2.73	95.21±5.13	#0.080
Total Energy Dissipation (µJ)	663.06±43.53	712.30±89.19	*0.252
**AFM**	**Normal (n = 5)**	**Cy/+ (n = 4)**	**p-values**
Indentation Elastic Modulus (MPa)	962.99 345.98	996.73 588.99	0.920

Values are presented as mean ± standard deviation.

*equal variance t-test.

# Wilcoxon ranked-sum test.

p-values less than 0.05 are in bold.

### AFM-based indentation

**Figure 4:**
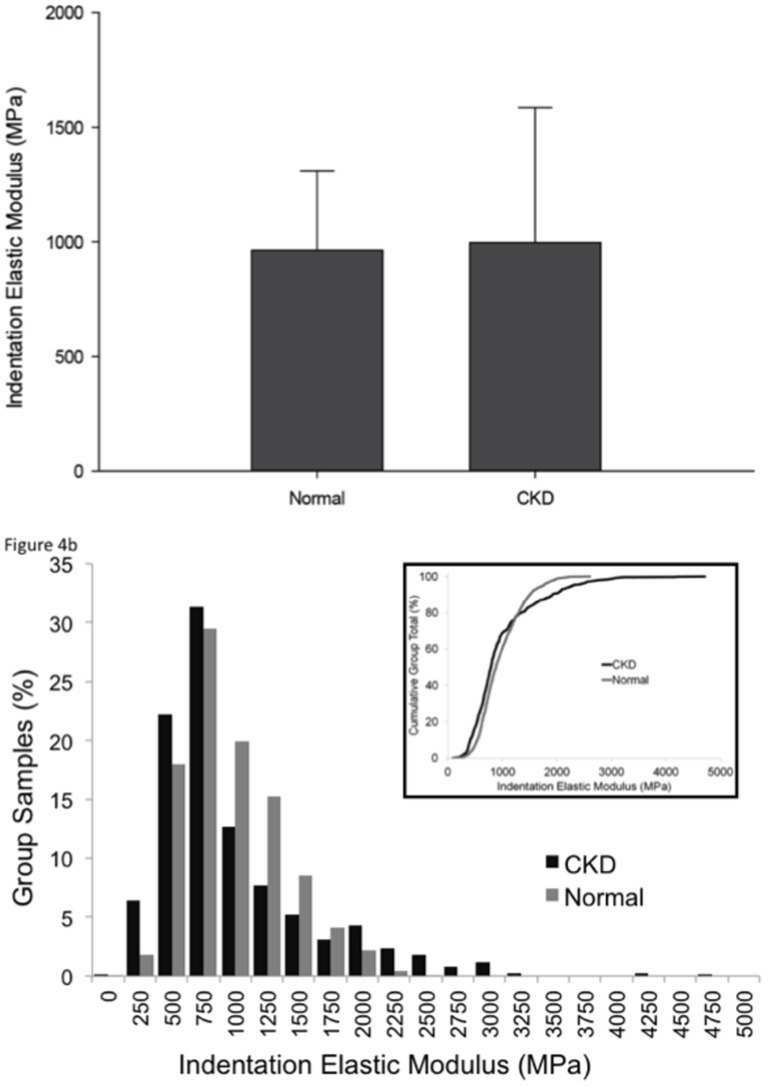
Nanoindenation reveals that CKD skeletal tissue has increased heterogeneity in the elastic modulus compared to normal bone. There was no significant difference in the average elastic modulus (A), but the distribution of elastic modulus values was significantly broader (B), with a greater proportion of both high and low values in CKD relative to normal (p<0.0001).

There was no difference in the indentation elastic modulus between the groups (**FIGURE 4a**). However, when all indentations within each groupwere considered as a population, the distribution of elastic modulus values did differ between the two groups. Animals with CKD had a greater proportion of both high and low values of elastic modulus than their normal counterparts (**FIGURE 4b**).

### Tissue Composition

Raman spectroscopy revealed no differences between animals with CKD and their normal counterparts with regard to overall compositionof the mineral and organic matrices (**Table 2**). Specifically, there were no differences between the groups in the phosphate-to-amide I ratio (the mineral-to-matrix ratio), mineral crystallinity, or the carbonate-to-phosphate ratio (a reflection of type B carbonate substitution).

**Table 2:** Tissue composition and collagen morphology.

**Raman**	**Normal (n = 6)**	**Cy/+ (n = 6)**	**p-values**
Crystallinity (1/FWHM PO_4_^3-^ν1)	0.054±0.001	0.055±0.001	*0.4421
Carbonate Substitution (CO_3_^2-^ν1/PO_4_^3-^ν1)	0.618±0.037	0.608±0.019	#0.5375
Relative Mineralization (PO_4_^3-^ν1/Amide I)	2.539±0.253	2.699±0.317	*0.9372
**AFM**	**Normal (n = 6)**	**Cy/+ (n = 5)**	**p-values**
D-Periodicity (nm)	65.641±0.646	65.864±0.838	*0.631
**HPLC**	**Normal (n = 8)**	**Cy/+ (n = 6)**	**p-values**
Pyridinoline per Collagen (mol/mol)	0.60±0.17	0.54±0.10	**0.252
Deoxypyridinoline per Collagen (mol/mol)	0.37±0.07	0.37±0.04	**0.974
Pentosidine per Collagen (mol/mol)	878± 153	921±148	*0.523

Values are presented as mean ± standard deviation.

*equal variances t-test.

**unequal variances t-test.

# Wilcoxon ranked-sum test.

### Collagen Morphology and Cross-Linking

There were no mean differences in D-periodicity between the two groups (**Table 2**). There were also no differences in the distribution of D-periodicity when all fibrils were considered. In addition, both enzymatic and non-enzymatic cross-linksas assessed by HPLC were similar between groups (**Table 2**).

## Discussion

The mechanical integrity of skeletal tissue is determined by the amount of tissue present, its distribution, and its quality. Compromises in any of these factors can lead to an increased fracture risk. The notable loss of cortical bone mass associated with CKD is assumed to be primarily responsible for the increased fracture risk seen in patients [5]. The current study advances our understanding of bone fragility in CKD by showing that microscale and nanoscale mechanical properties are alteredindependently of changes in bone mass and porosity.

Our lab has previously documented reductions in both structural and estimated materialproperties in Cy/+ rats, animals with progressive CKD [12], [32]. But, whole bone testing only provides indirect estimates of material properties, which is why wedirectly assessedmicroscale mechanical propertiesusing reference point indentation (RPI).RPI data from the present study indicate that animals with CKD had higher IDI, higher first cycle creep indentation distance, and first cycle energy dissipation. Taken together, these results indicatethat the tissue in animals with CKD is less resistant to indentation and more prone to damage. Indentation distance increase was nearly 20% higher in CKD animals compared to their normal counterparts. Similar differences in IDI have been previously reported in diabetic rats[33]. These data have two important implications. First, they show that CKD negatively affects skeletal tissue independently of bone mass, which means that estimates of bone massalone likely underestimate the overall mechanical effects of CKD.These differences in bone quality may explain the conflicting data available on BMD and fracture risk in CKD patients[6], [34]–[40]. Second, our data provide a basis for considering *in vivo* applications of RPI in the clinical setting of CKD. RPI has been used to successfully differentiate patients with and without hip fractures[41] as well as those with and without atypical femoral fractures [42]. A related indentation device has also been shown to discriminate patients with diabetes from their normal counterparts[43]. Assessment of tissue-level mechanical properties, combined with standard imaging modalities to measure bone mass, cortical geometry (especially porosity), and trabecular architecture, may prove to be an ideal combination by which to assess the overall mechanical integrity of bones in patients with CKD.

The current study also employed a hierarchical approach by examining nanoscale mechanical properties with atomic force microscopy. AFM indentation provides a direct assessment of the nanoscale stiffness produced by the collagen and mineral composite. Consistent with measurements of microscale stiffness (first cycle unloading slope), these results show that the average indentation elastic modulus was not significantly different between the two groups.Yet, the distribution of elastic modulus values was different. Animals with CKD displayed a greater degree of heterogeneity in nanoscale elasticity. Because increased material heterogeneity is often considered advantageous [44], these results may reflect an adaptive response to declining integrity at larger length scales. Alternatively, extreme variations in nanoscale properties maylead to localized stress concentrations that result in damage accumulation from lower forces[45]. Although heterogeneity is likely necessary for normal mechanical integrity, extreme heterogeneity may be problematic from a mechanical standpoint [46]. Future studies should attempt to better understand the role of material heterogeneity in CKD in order to specifythe contributionofmicroscale and nanoscalepropertiesto whole bone mechanical properties.

While whole bone, microscale, and nanoscale mechanical differences are present in animals with CKD, tissue composition, collagen morphology, and collagen cross-linking fail to account for the differences. These data conflict with studiesshowing higher mineral-to-matrix ratios, lower mineral crystallinity, increased advanced glycationendproducts (AGEs), and decreased gene expression of lysyl oxidase in alternative models of CKD [14]–[15], [47]. One potential explanation for these disparate results is that these previous studies utilized younger animals that developed advanced CKD during skeletal growth. Teasing apart the interaction between growth and disease is difficult, which is why the present study employed the use of a model in which kidney disease occurs after skeletal maturity.

Previous studies have demonstrated that non-enzymatic cross-links (pentosidine, specifically) are increased in the circulation of patients with CKD[48]–[51]. These findings coincide with the accumulation of AGEs in soft tissues detected by fluorescence methods[52]. Because high levels in the circulation are associated with the deposition of AGEs in other tissues, this may be true of skeletal tissue as well. To date, this has only been confirmed in one small clinical study in patients on dialysis [53]. Currently, there are few data in animal models, and clinical trials examining predialysis patients are lacking. Using a low turnover 5/6 nephrectomy model, two studies have reported increased pentosidineusing Raman spectroscopy[14]–[15]. While Raman spectroscopy has been utilized to detect AGEs in ocular tissue[54], its ability to detect changes in bone is unknown as HPLC is the standard method used to measure AGEs in skeletal tissue[53], [55]–[58]. Here, having employed HPLC, thehypothesized increases in AGE content were not observed. While increases may occur with more advanced disease in theseanimals, the relationship between circulating pentosidineand its accumulation in bone collagen in CKDremains unresolved.

At present, clinical data on bone quality in CKD are minimal. Aside from the aforementioned dialysis study [53], CKD patients with high bone turnover had reduced stiffness and a decreased mineral-to-matrix ratio as assessed on iliac crest biopsies [59]. As these data are from cancellous bone, though, direct comparison with the current work on cortical bone is difficult. Connecting these dots by examining cortical bone properties in patients (which is possible with iliac crest biopsies) and cancellous properties in rats will be an essential step in moving forward.

Limitations to the current study should also be recognized. First, because tissue from a previous study was used, the sample sizes used here were small. Hence, any applications to other animal models or patients should occur with a measure of caution. Second, the mechanical assessments were localized to the periosteal surface, which may not be fully representative of the entire cortex. As such, the assessed parameters may differ at other cortical sites. Finally, this study assessed material properties in 30-week-old animals. As the disease progresses, differences in composition and morphologymay arise.Nevertheless, the advantage of using 30-week-old animals is that these animals are skeletally mature but do not yet exhibit the rampant increase in cortical porosity present at 35 weeks [32].

In conclusion, these data show that both microscale and nanoscale cortical bone material properties are altered in an animal model of CKD. The specific skeletal tissue alterations responsible for these mechanical differences remain unclear. Nevertheless, in addition to bone loss and cortical porosity, defects in material-level mechanical properties may also contribute to the increased fracture risk associated with CKD.
